# Development of a tool to assess environmental factors to support home care – a Delphi study

**DOI:** 10.1186/s12877-023-04207-3

**Published:** 2023-08-21

**Authors:** Chloé Schorderet, Caroline H.G. Bastiaenen, Robert A. de Bie, Marc Maréchal, Noémie Vuilleumier, Lara Allet

**Affiliations:** 1 School of Health Sciences, HES-SO Valais-Wallis, University of Applied Sciences and Arts Western Switzerland, Valais, Sion, Switzerland; 2 Department of Epidemiology, Care and Public Health Research Institute CAPHRI, Maastricht University, Maastricht, the Netherlands; 3 The Sense Innovation & Research Center, Lausanne and Sion, Switzerland; 4 Neuchâtel Lung Association, Neuchâtel, Switzerland; 5 Department of Community Medicine, University Hospitals and University of Geneva, Geneva, Switzerland

**Keywords:** Environmental factors, Home care, Environment, Assessment, Delphi

## Abstract

**Background:**

Living in an adequate environment suited to one’s abilities and needs is an essential condition to function in daily life. However, no complete tool currently exists to provide a rapid overview of a person’s environment, both material (accommodation and auxiliary means) and social (entourage and available services). Our aim was to develop a tool to identify potentially problematic environmental factors and to determine when an in-depth assessment is necessary.

**Methods:**

Health professionals experienced in home-based treatment participated in a three-round Delphi process. The first round aimed to define which items the tool should contain, the second to collect participants’ opinions on a first version of the tool, and the third to collect the participants’ opinions on the adapted version of the tool.

**Results:**

A total of 29 people participated in the first round, 21 in the second and 18 in the third. The final tool contains 205 items divided into four categories (basic information about the inhabitant and their home, inhabitant’s level of independence and autonomy, home, tools and means at the inhabitant’s disposition) and two annexes (stairs to access to the home, internal staircase to the dwelling).

**Conclusions:**

A complete tool allowing professionals working in patients’ homes to obtain an overview of the environmental factors that could represent obstacles to the independence of the inhabitant, or to the possibility of providing quality care could be developed. This tool is very complete but relatively long. To facilitate its usability, it would be relevant that a digital version to focus on individual relevant categories be elaborated.

**Supplementary Information:**

The online version contains supplementary material available at 10.1186/s12877-023-04207-3.

## Introduction

The number of older people has been increasing steadily worldwide for many decades and this phenomenon is expected to continue in the coming years [1]. While life expectancy has increased over the past centuries [2], healthy life expectancy did not increase in the same way [3], which means that people live longer but suffer from co-morbidities. Indeed, the prevalence of chronic and degenerative diseases increases with advanced age [4, 5]. The number of dependent older people requiring long-term care will therefore certainly increase in the coming years [6]. In this context, optimal management of chronic diseases and multimorbidity is one of the main challenges facing the Swiss health system [7]. To address this challenge, a transition from institutional to home care seems to be relevant [8]. This type of care allows patients to be treated in a known and familiar place [9]. Moreover, it also avoids the negative consequences of hospitalizations [9] which are not always well tolerated by patients and can lead to an increase of disability and functional decline [10–13]. Finally, home care can also be economically relevant as its costs are generally lower than that of hospitalizations [14, 15].

In order to provide optimal care at home, a comprehensive patient-centred approach is recommended [16–18]. Such an approach requires instruments that assess both the health status and the context in which the individual functions. Regarding health and functional status assessment tools, the Resident Assessment Instrument-Home-Care (RAI-HC) [19, 20] is frequently used in Swiss home care and contributes to improving the quality of care provided to homecare recipients [21]. However, there are only a few tools that allow a standardised and comprehensive assessment of the environment. An example is the SAFER Home which is frequently used by occupational therapists [22]. However, this questionnaire cannot be used by the whole diversity of health professionals visiting the patient’s home, as it requires specific skills in task observation and activity analysis [16, 17]. Currently, therefore, there is no tool specifically developed to be used by all health professionals that is systematically integrated into home care in Switzerland. This makes outlining a policy and decisions about the possibility of home care and home maintenance challenging.

The fact that the environmental components are not systematically assessed is surprising since it plays a key role in an individual’s functioning. To illustrate this, the International Classification of Functioning, Disability and Health (ICF) [23], which was published in 2001, no longer defines disability as an attribute of the individual, but considers their functioning to depend on dynamic interactions between their health condition and the context in which the individual lives [24]. This classification differentiates between environmental factors that have facilitating effects and those that may represent barriers in the individual’s life [23].

Lawton and Simon [25] also highlighted the influence of the physical and social environment on the individual’s capacities. According to these authors, the more limited the capacities of individuals, the more supportive the environment should be [25]. In addition, it has also been shown that environmental factors such as housing, nuisance (related to social context and insecurity), residents, and neighbourhood have an influence on frailty and on performing activities [26], but also that the physical configuration of the environment has a determining role in social participation [27]. Finally, it has also been reported that an environment that meets individual needs, for example through the implementation of home adaptations, has positive effects on quality of life, independence, autonomy, and functional capacities of individuals concerned [28–33]. In addition, home adaptations also reduced fear of falling [34] and fall-related injuries [35], which has direct consequences on the healthcare system.

It is now widely recognised that environmental factors can either facilitate or hinder functioning of a person and performance of their activities of daily living. It is therefore essential that the environment is thoroughly assessed when providing home care. To this end, the development of a tool to assess environmental factors in a standardised way, i.e., the home itself (e.g., access to the home and characteristics of the various rooms) as well as the auxiliary means, the domestic appliances, and the assistive facilities, and of course the social aspects and the services and assistance available, is required.

The aim of this study was therefore to create consensus about a tool that could be used by all health professionals providing treatment in the patient’s home to assess environmental factors and to highlight those that might represent barriers to the person’s independence, or to the possibility of providing quality care.

## Methods

### Design

**Fig. 1:**
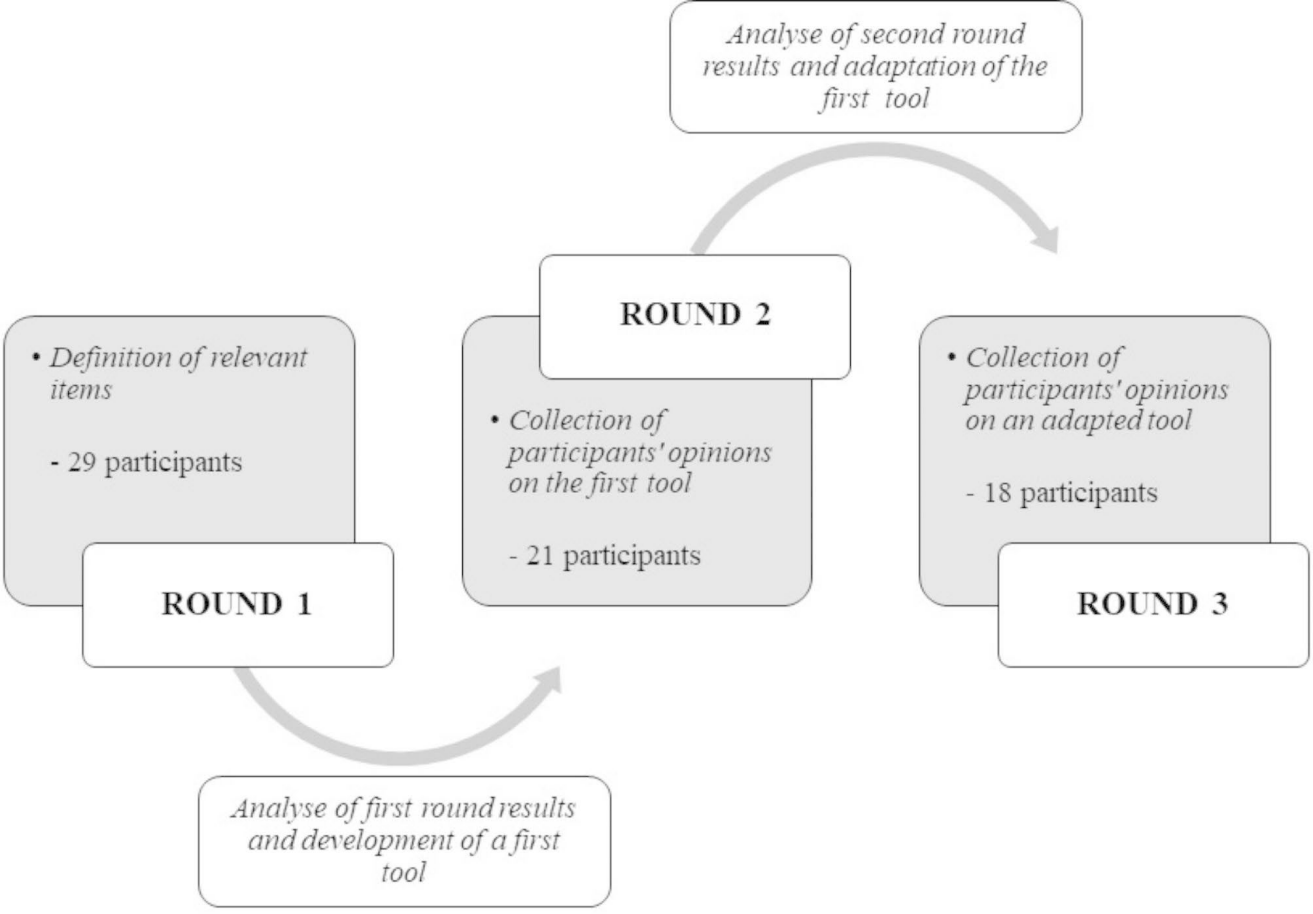
Representation of the of the different steps of the Delphi procedure

To achieve this objective, we conducted a Delphi study. This type of study allows for the gathering of opinions from experts through a series of iterative questionnaires, with the aim of reaching a group consensus [36]. This method is therefore ideal for developing a checklist tool, as it allows experienced people to give their opinion on the items to be included. This approach was already used by several authors to develop checklists in different fields [4, 5, 9]. The Delphi process was conducted in three different rounds until data saturation has been reached. The first round aimed to define the relevant items to be assessed in the tool; the second round aimed to collect the participants’ opinions on a first tool (developed according to the results of the first round); the third round aimed to collect the participants’ opinions on an adapted tool (adapted according to the results of the second round) (see Fig. 1). Regarding ethical considerations, the study did not fall under the purview of the Swiss Federal Human Research Act (LRH) and therefore did not require a request to the ethics commission. The study was carried out in accordance with Swiss legislation, the Declaration of Helsinki [37], and Good Clinical Practice guidelines.

### Participants

A variety of professionals with experience in home care from different regions of French-speaking Switzerland were recruited to participate in the study. People who experienced a home adaptation were also recruited. The participants were recruited mainly by sending emails to the home care agencies in the different cantons of French-speaking Switzerland. The contact persons of the agencies were informed about the aims of the study and its procedure and were invited to send us with permission, the contacts of their collaborators interested in participating in the study.

In addition, various individuals with experience in home care identified through internet searches were also contacted by email by the research team. For health and social care professionals, the inclusion criteria were to have at least a bachelor’s degree in health or social care (or a qualification recognized as equivalent), and to have at least one year’s experience in home care. For building professionals (such as home adaptation technician), the inclusion criteria were to have at least a federal patent and to have at least one year’s experience in home care. Also, persons who have experienced adaptations to their own home (i.e., an architectural modification of the home to improve the person’s independence) were included. According to the literature, a sample size of 10 to 15 participants suffices if the group is sufficiently homogeneous [38]. In our study, the following five categories of participants were defined: (1) nurses, (2) occupational therapists, (3) physiotherapists, (4) others (doctors, psychologists, social workers, building professionals), (5) people who experienced a home adaptation. The aim was to recruit at least three people per category so that all professionals working in home care as well as people who had experienced home adaptations were represented. People in category 5 (people who had experienced home adaptations) were invited to participate only in the first round because the questions in the second and third rounds were specifically intended for users of the tool (professionals working in home care).

### Data collection

In each round, data were collected through a questionnaire developed using REDCap software (REDCap 12.4.16 - © 2022 Vanderbilt University). The link for the questionnaire was sent by email. Prior to data collection, the online questionnaire for each round was tested by at least three individuals not involved in the study (healthcare professionals experienced in research on older adults or specialized in home care) and was adapted where necessary.

### Round 1

To prepare for the first Delphi round, a literature search was performed, and semi-structured interviews were conducted with the aim of creating a list of items to be assessed. The literature search was performed on the following databases: Pubmed, Cinahl, Embase and Cochrane. The research allowed the identification of 28 existing questionnaires assessing the environment. Items included in these questionnaires were listed and classified into eight categories: (1) basic information, (2) access to the home, (3) essential rooms in the home (kitchen, bathroom, bedroom, interior staircase), (4) additional rooms (hall, living room, laundry, cellar, garage, balcony / terrace), (5) functional aids, (6) various, (7) social aspects, (8) functional independence. Most categories contained sub-categories which are presented in Table 1. The item list was then completed by the responses obtained during the semi-structured interviews. These interviews were designed to highlight (i) how environmental factors were assessed and which ones were relevant to consider, (ii) what auxiliary means and assistive facilities were proposed to patients, (iii) how patient needs and safety were assessed, and (iiii) what were the needs of professionals working in home care related to environment assessment. Nine interviews were conducted. Three nurses, an occupational therapist, a physiotherapist, a psychologist, an architect, a technician specialized in home adaptations, and a person who had benefited from a home adaptation were interviewed.

The final list consisted of 259 items that participants were invited to rate in the first Delphi round, using a 5-point Likert scale (“not at all important to assess,“ “not important to assess,“ “neither important nor not important to assess,“ “important to assess,“ “very important to assess”). The number of items per category and subcategory is presented in Table 1. At the beginning of the first round, professionals working in home care were also asked to indicate their age, occupation, number of years of experience in the profession, number of years of experience in home care, level of education, and how long maximum the final questionnaire should take to complete. Individuals who had experienced home adaptations were asked to indicate their age, whether their home had been modified to improve their independence, whether they used auxiliary means to improve their independence, and how long maximum the final questionnaire should take to complete. At the end of each category, participants could suggest new items for that category that they considered relevant for assessment. Finally, at the end of the questionnaire, participants had the possibility to add overall comments.

Participants were given about three weeks to complete the questionnaire. Their responses were exported from REDCap into Excel software for analysis. Items rated as “important to assess” or “very important to assess” by at least 67% of participants were retained for development of the new tool [39]. New items suggested by participants and comments from them were discussed by the research team (which was made up of health professionals with experience in research on older adults and environment) and retained according to their relevance. In cases where it was difficult to reach a consensus among the members, the comments concerned were discussed with an expert panel made up of professionals specializing in home care. Based on the selected items and comments, a first version of the tool was then created. Before being sent to the participants for the second round, this version was reviewed and approved by all research team members.

**Table 1:** Categories, sub-categories and numbers of items submitted to the participants in the first round

Categories	Sub-categories	Number of items
1. Basic information	-	4
2. Access to the home	Vehicular access to the building/house	2
	Exterior: access to the main entrance of the building/house	11
	Entrance to the main building	8
	Access to the home (to the flat itself)	16
3. Essential rooms in the home	Kitchen	19
	Bathroom	32
	Bedroom	25
	Interior staircase	15
4. Additional rooms	Entrance hall	10
	Living room	15
	Laundry	16
	Cellar	14
	Garage	14
	Balcony / terrace	6
5. Functional aids	Assistive devices	4
	Communication tools	6
	Tools to support activities of daily living	9
6. Various	-	5
7. Social aspects	Social context	5
	Services in the neighborhood	6
	Leisure	6
8. Functional independence	-	11
Total		**259**

### Round 2

In the second round, participants received a summary of the first-round results and the first version of the tool. They were then asked to indicate if they had any comments regarding the introduction of the tool. Subsequently, for each item category, participants were asked to indicate in a binary manner (yes or no) whether the instructions were comprehensible, whether all items were comprehensible, whether all items were relevant, and if they felt able to answer all items. If they answered “no” to any of these questions, they were asked to indicate which item(s) were concerned and to justify their answer. In addition, for each item category, they were asked to indicate in a binary manner (yes or no) if they considered any items to be missing. If they indicated “yes,“ they could then suggest new items. At the end of each category, participants also could provide comments.

Participants had about three weeks to complete the questionnaire. Afterwards, responses were exported to Excel for analysis. For each category, instructions/items assessed as comprehensible/relevant by more than 67% of participants [39] were considered as such and retained without modification. For each category, items that more than 67% of participants indicated they felt able to answer were retained without modification. For each category, if less than 67% of participants indicated that no items were missing, proposals for new items were added to the tool. These proposals were based on participants’ suggestions. Participants’ comments were discussed by the research team and integrated to the tool according to their relevance. The analysis of the data from the second round resulted in the adaptation of the first version of the tool. Before being sent to the participants for the third round, the adapted tool was reviewed and approved by all research team members.

### Round 3

In the third round, participants received a summary of the second-round results and an adapted version of the tool to download. In this version, the parts that were modified according to the second-round results were highlighted with color so that participants could see the changes. Participants were then asked to indicate in a binary manner (yes or no) if they had any comments on the modified parts. If they answered yes, they were invited to write their comments. They were also asked to assess the relevance of the new items using the same 5-point Likert scale as in the first round. Participants were then asked to indicate how much time they estimated it would take to use the tool. They were also invited to indicate in a binary manner (yes or no) whether they felt the questionnaire was usable in practice. If they answered no, they were asked to indicate why. Finally, participants were invited to suggest a title for the environmental assessment tool.

Participants were given about three weeks to complete the questionnaire. Afterwards, responses were exported to Excel for analysis. Regarding the modified parts, participants’ comments were discussed by the research team and integrated into the final tool according to their relevance. For the new items, those rated as “important to assess” or “very important to assess” by at least 67% of participants were retained for the final tool [39]. For the rest of the analysis, the average was calculated for the estimated time to use the tool and the frequency was reported for the usability of the questionnaire in practice. The analysis of the data from the third round resulted in the drafting of the final tool.

## Results

### Round 1

In total, 29 people agreed to participate in the study. Of these, 28 completed the questionnaire for the first round (26 completely and 2 partially). The characteristics of the first-round participants are described in Table 2. Regarding the maximum time the final tool should take to complete, all participants responded and indicated that the duration should not exceed 24 min (SD = 15).

Of the 259 items assessed by participants, 200 were rated as “important to assess” or “very important to assess” by more than 67% of participants. In addition, participants proposed 70 new items to be added to the tool and drafted nine comments.

The selected items and the participants’ comments led to a modification of the tool’s structure. The following five categories were defined: (1) basic information about the inhabitant, (2) functional independence of the inhabitant, (3) tools and means at the inhabitant’s disposition, (4) context, (5) home (access to the home, kitchen, bathroom, bedroom, interior staircase, entrance hall, living room, laundry, cellar, garage, balcony / terrace). In the “home” category, for the kitchen, bathroom, bedroom and interior staircase, two types of items were to be evaluated. The first type of items concerned the evaluation of the environment itself, and the second type concerned the evaluation of the auxiliary means, domestic appliances, and assistive facilities. The appendix “exterior staircase” and the appendix “interior staircase to access to the home” were added to the tool.

Regarding the responses of people whose home was adapted, two indicated that their home was modified to improve their independence and one indicated that it was adapted for another reason (without specifying which one). Regarding the use of auxiliary means, two people reported using them to improve their independence and one specified using an electric wheelchair, a door electrification system, and an elevator.

**Table 2:** Characteristics of first round participants

	Professionals working in home care (n = 25)	People who benefited from a home adaptation (n = 3)
Gender	Women : 17 Men : 8	Women : - Men : 3
Age	40.2 (SD = 9.5)	71 (SD = 6)
Profession	Nurses: 8 Occupational therapists: 8 Physiotherapists: 4 Psychologist: 1 Social workers: 3 Home adaptation technician: 1	-
Number of years of experience in the profession	13.8 (SD = 8.1)	-
Number of years of experience in home care	7.7 (SD = 5.5)	-
Professional background	Federal patent: 1 Bachelor’s degree: 16* Master’s degree: 8	-

* Of these 16, one occupational therapist and one physiotherapist did not have a bachelor’s degree, but had a qualification recognized as equivalent.

### Round 2

A total of 21 people participated in the second round. These included four nurses, eight occupational therapists, four physiotherapists, one doctor (who did not participate in the first Delphi round), one psychologist, two social workers, and one home adaptation technician. The structure of the tool (categories, sub-categories and number of items related to each one) which was submitted to the participants for the second round is presented in Table 3.

All instructions and items were rated as comprehensible by more than 67% of participants. All items were rated as relevant by over 67% of participants. For each item, more than 67% of the participants indicated that they felt able to answer it. Regarding missing items, less than 67% of the participants indicated that there were no missing items for the following five categories: “basic information about the inhabitant”, “functional independence of the inhabitant”, “tools and means at the inhabitant’s disposition”, “bedroom”, “interior staircase”. According to the participants’ suggestions, ten new items concerning these categories were therefore proposed to the participants during the third-round.

In addition, 129 comments were written by the participants. These comments allowed clarification of different instructions and items. They also led to a change in the structure of the questionnaire. The category “basic information about the inhabitant” was renamed “basic information about the inhabitant and their home” and several items that were in the category “context” were moved to this category. The category " inhabitant’s functional independence " was replaced by the category “inhabitant’s level of independence and autonomy”. The category “context” was deleted and the items it contained were divided into the categories “basic information about the inhabitant and their home” and " inhabitant’s level of independence and autonomy “. Several items from the category “access to the home” were moved to the category " inhabitant’s level of independence and autonomy “. In addition, a space for the assessor to write a comment or clarification was added at the end of each category.

**Table 3:** Structure of the tool submitted to the participant for the second round

Categories	Sub-categories	Number of items
1. Basic information about the inhabitant	-	5
2. Functional independence of the inhabitant	-	11
3. Tools and means at the inhabitant’s disposition	-	10
4. Context	-	17
5. Home	Access to the home	14
	Kitchen	16
	Bathroom	27
	Bedroom	16
	Interior staircase	7
	Entrance hall	6
	Living room	11
	Laundry	10
	Cellar	7
	Garage	6
	Balcony / Terrace	3
Annex I: External staircase		6
Annex II: Internal staircase to the dwelling		7

### Round 3

A total of 18 people participated in the third round. These included four nurses, three physiotherapists, seven occupational therapists, one psychologist, two social workers and one home adaptation technician. The structure of the tool (categories, sub-categories and number of items related to each one) which was submitted to the participants for the third round is presented in Table 4.

Only one participant commented on the changes made to the questionnaire after the second round. He suggested that the vocabulary used in the category “basic information about the inhabitant and their home” should be standardized for the items related to people providing assistance. This comment was addressed, and the tool was adapted accordingly.

Regarding the ten proposals of new items, eight were rated as “important to assess” or as “very important to assess” by more than 67% of participants and were therefore added to the tool. Given the small number of items retained after the third round, the authors considered that data saturation had been reached. The final draft of the tool is presented in Appendix I.

Regarding the duration of the questionnaire, the participants estimated that it would take 51.4 min to complete (SD = 27.9).

About the feasibility of the questionnaire, 16 people answered that the questionnaire seemed usable in their daily practice and two answered that it was not. Of these, one indicated that the content was adequate, but the format was problematic, and the other indicated that the questionnaire was too long and detailed to be used in daily practice.

**Table 4:** Structure of the tool submitted to the participant for the third round

Categories	Sub-categories	Number of items
1. Basic information about the inhabitant and their home	-	12
2. Inhabitant’s level of independence and autonomy	-	24
3. Home	Access to the home	11
	Kitchen	16
	Bathroom	27
	Bedroom	16
	Interior staircase	7
	Entrance hall	6
	Living room	11
	Laundry	10
	Cellar	7
	Garage	6
	Balcony / Terrace	3
4. Tools and means at the inhabitant’s disposition		10
Annex I: Stairs to access to the home		8
Annex II: Internal staircase to the dwelling		8

## Discussion

The three rounds of this Delphi study allowed the development of a tool to assess environmental factors and to highlight those that might represent barriers to the person’s independence, or to the possibility of providing quality care. This tool was developed based on reflections and proposals from professionals with varied and complementary backgrounds, all experienced in home care. We consider it to be a valuable basic tool to be used by all professionals working in patients’ homes. In the final round, the large majority of participants (n = 16: 90%) indicated that they found the tool useful for their practice. However, it is important to note here that the purpose of this tool was to highlight environmental factors that could be problematic, and not to assess the home in detail to propose specific adaptations. To this end, it is relevant that the tool is used in the first visit to the patient’s home so that environmental factors can be integrated from the beginning in the considerations about care and the possibility of home maintenance. In cases where the tool shows that complex adaptations are needed, specific assessments should be organized. These should be performed by professionals specialized in this field, such as occupational therapists. The latter have highly developed skills in assessing and adapting environmental factors that impact on people’s participation, and also in assessing and adapting assistive technologies [40].

Our literature search to develop the first round of the Delphi highlighted the existence of many questionnaires to assess the environment [41–48]. However, we did not find any that assessed the environment per se (home and its fittings), the social context, and the auxiliary means and assistive facilities available to the inhabitant all together. The SAFER-HOME seemed to be an adequate tool for assessing safety at home [22], however, it is relatively time-consuming to use (about one hour) [49] and is intended to be completed by occupational therapists only [22]. In this context, we consider our tool a good complement to existing tools since, on the one hand, it assesses the environment in a global way (the home itself, but also the context and the auxiliary means/assistance facilities available), and on the other hand, it can be used by all health professionals carrying out treatments in patients’ homes. We find this to be a key issue, as many patients are treated at home by professionals without specific training in environmental assessment and adaptation, such as nurses, physiotherapists, or psychologists. Given the importance of the environment on patient participation [26, 27, 50], it is essential that all professionals involved in home care can identify potentially problematic environmental factors. Our tool aims to meet this objective but also to highlight when an in-depth assessment is needed and when a professional specializing in this area, for example an occupational therapist [22], should be contacted.

Regarding the methodology, the authors decided to consider that there was agreement between the participants if at least 67% of the group expressed the same judgement, as it has been done in other studies aimed at developing a checklist [39, 51]. It would have been possible to consider agreement only at 80%, as is sometimes reported in the literature [52–54]. However, this would have led to a shorter questionnaire as we would have excluded a few more items. Given that the aim was to develop a comprehensive questionnaire, the authors considered the 67% threshold to be relevant. In addition, it’s important to mention here that there is currently no clear consensus on the agreement rate for Delphi methods, and that this depends on the research question and topic [38, 55].

A major strength of our study is the large number of participants. The fact that 28 people participated in the first round, 21 in the second, and 18 in the third allowed for many different opinions and reflections. These were particularly complementary as they came from professionals with a wide range of backgrounds. Another strength is that participants had significant experience in home care (on average 7.7 years for participants in the first round).

A first limitation relies in the first round of the study. During this one, participants added many new detailed items which needed specific competencies of their profession. To remedy this, our aim was reclarified to participants at the beginning of the second round and we emphasized that the purpose of the tool was that any health professional is able to identify environmental factors that might be problematic and also to indicate when the intervention of a specialized professional, such as an occupational therapist, was necessary.

An additional limitation concerns the Delphi method itself. The fact that a consensus has been reached does not guarantee that the items benefiting from the consensus are the most relevant [36]. It is therefore important to mention here that the tool is not yet validated, and that it is only a consensus of experienced people in a first step. The aim, nevertheless, is not yet to obtain a validated questionnaire, but rather a qualitative overview of environmental factors, with a specific view to implementing adaptations or involving a specialist if necessary. One further small drawback is that there was no direct interaction between the participants. However, this limitation needs to be balanced, as participants were relatively unanimous in their responses, and the agreement rate for most items was over 67%. Direct interactions would certainly not have impacted much on the results. Another concern is the fact that one doctor participated only in the second round, and one nurse and one social worker participated only in the first and third rounds. This is regrettable, since the purpose of the Delphi process is for ideas to develop from one round to the next [36]. The inclusion of these people even though they had not participated in all three rounds was discussed by the research team, who considered their participation relevant. In order to balance the fact that these people did not take part in the three rounds, the research team was particularly vigilant in the explanations of the results and of the questionnaire development process at the beginning of each round. A further limitation concerning the participants is the fact that no women nor people with other gender orientation were present in the group that benefited from a home adaptation.

Moreover, another drawback of this tool is that it is currently very long. This can make it difficult to use, both for the assessors, who have to invest part of their time in this task, but also for the inhabitants whose homes are being assessed. In the first round, participants indicated that the questionnaire should not take more than 24 min to complete, and in the third round, they estimated that the time for completion was 51.4 min. In addition, in the third round, two participants indicated that they felt the tool was not usable in practice, and one indicated that one of the reasons for this was that its length. The significant length of the tool is related to the fact that our aim was to have a comprehensive tool that could assess every room that a home might contain and all the environmental factors potentially present in the different rooms. In practice, however, the items to be assessed will depend on the overall context, and individual needs and aims, and the assessor will therefore be able to choose which parts of the questionnaire to use according to these criteria. In many situations, only certain parts of the tool will be used, and the assessment will therefore take less time to complete than the 51.4 min estimated by the participants.

In order to promote the use of the tool in practice, we consider that it would be relevant to develop a digital version of it. This would allow the assessor to choose the categories to be evaluated according to their relevance to the person concerned. For example, if the inhabitant would not have an entrance hall, the assessor could indicate it and all the items concerning this room would not appear in the tool. In addition, the aim for the digital version would also be to automatically generate a report of environmental factors which could represent barriers to the person’s independence, or to the possibility of providing quality care. This report would give the assessor an overview of the elements to be adapted. Furthermore, we plan that the digital version allows to get information about possible first and not invasive adaptations or redirect the latter to the appropriate people to contact, depending on the barriers identified. For example, if the assessor notes that a light switch is difficult to reach, the digital version could suggest in the final report that a motion-sensor bulb should be proposed to the inhabitant. In cases where specialist assessment is recommended, the digital version could, for example, directly propose the contacts of occupational therapists working in the relevant care network.

In parallel, the authors plan to develop an extra application that could be used directly by the inhabitants themselves and their informal caregivers. Such an application would have the advantage of enable the inhabitants to quickly get an overview of how they could adapt their environment to increase their independence and safety. To make this possible, the tool will first need to be adapted. Once both digital versions are developed, it will be relevant to conduct new studies to evaluate their usability.

## Conclusions

Home care has developed significantly in recent years in Europe [56] and the environment of people benefiting from this type of care plays a key role both in their independence and in the possibility for health professionals to provide quality care. In this context, it is essential that professionals providing home care can quickly assess it and the tool developed through this study should be used for this purpose. It will allow the assessor to quickly obtain an overview of the environmental factors that could represent obstacles to the independence of the inhabitant, or to the possibility of providing quality care. However, the current tool needs to be further developed to make it easier to use. To this end, the authors plan to develop a digital version.

### Electronic supplementary material

Below is the link to the electronic supplementary material.

Supplementary Material 1

## Data Availability

The datasets used and analyzed during the present study are available from the corresponding author on reasonable request.
