# Supplementary material for: Development of a tool to assess environmental factors to support home care – a Delphi study
Source: BMC Geriatr. 2023 Aug 21;23:501. doi: 10.1186/s12877-023-04207-3 (PMC10441718; doi:10.1186/s12877-023-04207-3)
Supplement: Supplementary file 1 — Supplementary Material 1 [file 12877_2023_4207_MOESM1_ESM.pdf]

APPENDIX I

Assessment of environmental factors to support home care

This tool is intended for all health professionals providing care in patients' homes. Its aim is to highlight the environmental factors that could represent obstacles to the independence and/or functioning of the inhabitant in his or her daily life, or to the possibility of providing care in adequate conditions.

It consists of the following parts:

1. Basic information about the inhabitant and their home .... page 2
2. Inhabitant's level of independence and autonomy.. page 4
3. Home .... page 6
   - 3.1 Access to the home .... page 7
   - 3.2 Kitchen .... page 8
   - 3.3 Bathroom .... page 9
   - 3.4 Bedroom .... page 11
   - 3.5 Interior staircase .... page 12
   - 3.6 Entrance hall page13
   - 3.7 Living room page14
   - 3.8 Laundry page14
   - 3.9 Cellar page15
   - 3.10 Garage .... page 16
   - 3.11 Balcony / Terrace .... page 17
4. Tools and means at the inhabitant's disposition .... page 18

Each part consists of different questions and/or tables in which you are asked to put a cross in the appropriate box. At the end of each section, you have the possibility to add remarks/clarifications.

For all questions, please answer **according to your judgement as a health professional** and not according to the feeling of the inhabitant.

Throughout the tool, the notion of independence refers to activities of daily living. When the term "independently" is used, it means that the inhabitant can do it alone. The term "safely" means that the inhabitant can perform the activity concerned without endangering him/herself or risking a fall.

Full name of the assessor: ....

Assessor's profession: ....

Date of assessment: ....

1. BASIC INFORMATION ABOUT THE INHABITANT AND THEIR HOME

1.1 Full name : ....

1.2 Age : ....

1.3 Gender :

- Woman
- Male
- Other

1.4 Medical diagnosis: ....

1.5 Main impairment(s) : ....

1.6 Home status :

- Owner
- Tenant

1.7 Type of accommodation :

- Flat
- Single-family house
- Other: ....

1.8 Presence of other persons living in the same home :

- No
- Yes

  If yes, number of people: ....

  Person 1:

  Name, first name and relationship to the inhabitant:

  ....

  Does person 1 provide assistance to the inhabitant in his or her daily life?

  - No
  - Yes

    If yes, what kind of help does person 1 provide to the inhabitant?

    ....

  Person 2:

  Name, first name and relationship to the inhabitant:

  ....

  Does person 2 provide assistance to the inhabitant in his or her daily life?

  - No
  - Yes

    If yes, what kind of help does person 2 provide to the inhabitant?

    ....

1.9 Presence of persons providing assistance not living in the same home :

- No
- Yes

  Person 1:

  Name, first name and relationship to the inhabitant:

  ....

  Type of assistance provided:

  ....

  Frequency of aid: ....

  Person 2:

  Name, first name and relationship to the inhabitant:

  ....

  Type of assistance provided:

  ……………………………..

  Frequency of aid: ....

1.8 Presence of a neighbor who can provide help in an emergency:

- No
- Yes

1.9 Inhabitant with social insurance (e.g., AHV, IV, social assistance):

- No
- Yes

  If yes, type of social insurance(s) received:

  ....

1.10 Inhabitant under tutorship :

- No
- Yes

  If yes, name and surname of the tutor: ....

1.11 Presence of a pet in the accommodation:

- No
- Yes

  If yes, type of animal: ....

1.12 Presence of difficulties related to specific winter weather conditions (e.g., presence of snow making it difficult to access the home):

- No
- Yes

  If yes, type of difficulty: ....

1.13 Presence of difficulties related to specific summer weather conditions (e.g., presence of extreme temperature in the home):

- No
- Yes

  If yes, type of difficulty: ....

1.14 Comment(s) / clarification(s) on the category "Basic information on the inhabitant and their home" :

....
....
....

2. INHABITANT'S LEVEL OF INDEPENDENCE AND AUTONOMY

2.1 - Table

For each activity listed, please put a cross in the appropriate box. You can also write a comment / clarification in the right column.

If you tick the box "receives help from a third party to do so", please indicate in the right column the frequency of the help received and the person/organisation providing this help.

	A. Is able to do so independently and safely	B. Receives help from a third party to do so	C. Requires in-depth assessment	D. Need for assistance to be organised	Comment / clarification
2.1.1 Getting up / sitting down / lying down[1]					
2.1.2 Dressing / undressing[1]					
2.1.3 Toileting					
2.1.4 Going to the toilet					
2.1.5 Eating					
2.1.6 Cooking					
2.1.7 Doing laundry					
2.1.8 Cleaning up					

2.2 Is the inhabitant able to move independently and safely?

- No
- Yes

2.3 Does the inhabitant use an aid to mobility (e.g., cane, rollator)?

- No
- Yes

  If yes, aid used: ....

  If yes, for which trip(s) is this aid used?

  - All travel on foot
  - Travel on foot outside the home
  - Other: ....

2.4 If the inhabitant uses an aid to get around, is it adequate (in good condition and adapted to the inhabitant's needs)?

- Yes
- No

[1] If only part of the activity can be carried out independently and safely, please specify this in the right column.

If not, why not: ....

2.5 Does the inhabitant own a car that he/she can drive?

- No
- Yes

If yes, does the inhabitant have a parking space in front of the building/house where he/she lives?

- Yes
- No

2.6 - Table

Please put a cross in the appropriate box. If you tick box "A. Yes", please indicate in the right column the means of transport used to get to the place concerned.

	A. Yes	B. No, but not a problem[2]	C. No. A solution must be put in place	D. If yes, means of transport used to get there
Social context				
2.6.1 Ability to travel independently to **family members** not living in the same home				
2.6.2 Ability to travel independently to **friends** who do not live in the same house				
Services in the vicinity				
2.6.3 Independent access to the **bank**				
2.6.4 Independent access to the **post office**				
2.6.5 Independent access to the **supermarket**				
2.6.6 Ability to travel independently to **health care providers** (e.g., doctor, dentist, physiotherapist, pharmacist)				
Leisure				
2.6.7 Ability to go independently to a **park/walking area**				
2.6.8 Possibility of independent access to **cultural spaces** (cinemas, theatres, libraries, etc.) of one's choice				
2.6.9 Independent access to **religious spaces of** choice				
Other				
2.6.10 Independent access to the **mailbox**				
2.6.11 Independent access to the **waste room**				

2.7 Comment(s) / clarification(s) on the category "Inhabitant's level of independence and autonomy" :

....

....

....

[2] This box can be ticked, for example, if the inhabitant does not wish to go to the place concerned or if he/she can go with the help of a third person.

3. HOME

This part of the tool assesses the different rooms in the home. Depending on the room, you will be asked:

- to answer questions
- to assess the environment itself (e.g., the width of doors)
- assess the aids (e.g., presence of grab bars), household appliances (e.g., oven) and equipment present (e.g., ramp on stairs)

To answer questions about how to carry out an activity, we invite you **to observe the inhabitant carrying it out.**

For the tables concerning the environmental assessment, you have the following answer options:

- **"Yes"** means that the item being assessed is adequate.
- **"No. A solution must be put in place**" means that the item assessed is not adequate and that a solution must be put in place.
- **"No, but not a problem"** means that the item assessed is not adequate but that this is not a problem for the inhabitant. For example, in the question "Is the inhabitant able to get in and out of the bathtub independently and safely?" you could tick "No, but not a problem" if the inhabitant is unable to get into the bathtub independently and safely but has a shower that he/she can get into safely.
- **"Requires in-depth assessment**" means that in order to answer the question, an in-depth assessment is required.
- **"Not applicable**' means that the item in question cannot be assessed. For example, you could tick this answer to the question "Is the inhabitant able to step over the bathroom threshold safely?" if the bathroom did not have a threshold.
- For each question, you also have the possibility to write a comment / clarification in the right column.

For the tables on aids, household appliances, and equipment present, you have the following answer options:

- **"Present and adequate"** means that the means/appliance/equipment is present, in good condition and suitable for the inhabitant.

  "Present but requires adaptation" meaning that the means/appliance/equipment is present but needs to be adapted or modified for the inhabitant to use it adequately.
- **"Not present and not needed"** means that the means/appliance/equipment is not present, but the inhabitant does not need it.
- **"Not present but needed and to be installed"** meaning that the means/appliance/equipment is not present, but the inhabitant would need it and therefore it should be installed.
- **"Not present but needed and requires a thorough assessment"** meaning that the means/appliance/equipment is not present, but the inhabitant would need it and its installation requires a thorough assessment by a competent professional.

3.1 Access to the home

3.1.2 On which floor is the accommodation located? ....

3.1.3 If the home is not on the ground floor, does the inhabitant have to use a staircase to get there?

- Yes,

  If yes, please complete Annex I (found at the end of the tool)
- No, the inhabitant can use a lift

  If the inhabitant can use a lift, is he/she able to do so independently and safely?
  - Yes
  - No

Table - 3.1.4

For each item, please put a cross in the appropriate box. You also have the possibility to write a comment / clarification in the right column.

	Yes	No. A solution must be put in place	No, but not a problem	Requires in-depth assessment	Not applicable	Comment / clarification
**Exterior: access to the main entrance of the building/home**						
3.1.4.1 Can the inhabitant access the main entrance of the building/home independently and safely?						
**Entrance to the main building** [3]						
3.1.4.2 Is the entrance to the main building sufficiently lit for the inhabitant to move around safely?						
3.1.4.3 Does the inhabitant manage the light sources in the main building entrance safely?						
3.1.4.4 Is the inhabitant able to open and close the main building entrance door independently and safely?						
3.1.4.5 Is the door to the main building wide enough to allow the inhabitant to pass through with an aid (such as a rollator or wheelchair)?						
3.1.4.6 Is the inhabitant able to step over the threshold of the main building independently and safely?						
**Access to the home (to the flat itself)**						
3.1.4.7 Is the front door of the home wide enough to allow the inhabitant to pass through with an aid (such as a rollator or wheelchair)?						
3.1.4.8 Is the inhabitant able to step over the threshold of the front door of the home independently and safely?						

[3] To be completed in case the inhabitant lives in a block of flats or in a house with several flats.

3.1.5 Comment(s) / clarification(s) on the category “Access to the home”:

....
....
....

3.2 Kitchen

Environment

Table - 3.2.1

For each item, please put a cross in the appropriate box. You also have the possibility to write a comment / clarification in the right column.

	Yes	No. A solution must be put in place	No, but not a problem	Requires in-depth assessment	Not applicable	Comment / clarification
3.2.1.1 Is the kitchen door wide enough to allow the inhabitant to pass through with an aid (such as a rollator or wheelchair)?						
3.2.1.2 Is the inhabitant able to step over the kitchen threshold independently and safely?						
3.2.1.3 Is the kitchen sufficiently lit for the inhabitant to use it safely?						
3.2.1.4 Is the kitchen space large enough for the inhabitant to move around freely, potentially with an aid?						
3.2.1.5 Is the kitchen space free of clutter (allowing the inhabitant to move around safely)?						
3.2.1.6 If there are mats in the kitchen, are they fixed to the floor to reduce the risk of falling?						
3.2.1.7 Is the inhabitant able to independently and safely access storage space for basic utensils (for basic cooking and eating)?						
3.2.1.8 Is all the furniture in the kitchen stable?						
3.2.1.9 Is the inhabitant able to use the hob safely?						
3.2.1.10 Is the electrical equipment in the kitchen (sockets and cables) safe?						

Household appliances

Table - 3.2.2

For each item, please put a cross in the appropriate box. You also have the possibility to write a comment / clarification in the right column.

	Present and adequate	Present but requires adaptation	Not present and not needed	Not present, but necessary and to be installed	Not present but necessary and needs further assessment	Comment / clarification
3.2.2.1 Refrigerator						
3.2.2.2 Oven						
3.2.2.3 Hotplates						
3.2.2.4 Dishwashers						
3.2.2.5 Freezer						

3.2.2.6 Microwave						

3.2.3 Comment(s) / clarification(s) on the category “Kitchen”:

....
....
....

3.3 Bathroom

Environment

Table - 3.3.1

For each item, please put a cross in the appropriate box. You also have the possibility to write a comment / clarification in the right column.

	Yes	No. A solution must be put in place	No, but not a problem	Requires in-depth assessment	Not applicable	Comment / clarification
**General information**						
3.3.1.1 Is the door to the bathroom wide enough to allow the inhabitant to pass through with an aid (such as a rollator or wheelchair)?						
3.3.1.2 Is the inhabitant able to step over the bathroom threshold independently and safely?						
3.3.1.3 Is the bathroom sufficiently lit for the inhabitant to use it safely?						
3.3.1.4 Is the inhabitant able to manage the light sources in the bathroom safely?						
3.3.1.5 Is the space in the bathroom large enough for the inhabitant to move around freely, potentially with an aid (such as a rollator or wheelchair)?						
3.3.1.6 Is the space in the bathroom large enough to accommodate a seating arrangement if necessary?						
3.3.1.7 Is the bathroom large enough for one person to help the inhabitant with their care in the bathroom if necessary?						
3.3.1.8 Is the bathroom space free of clutter (allowing the inhabitant to move around safely)?						
3.3.1.9 If there are mats in the bathroom, are they fixed to the floor to reduce the risk of falling?						
3.3.1.10 Is the inhabitant able to independently and safely access bathroom storage areas and the items they contain?						
3.3.1.11 Is all the furniture in the bathroom stable?						
3.3.1.12 Is the electrical equipment in the bathroom (sockets and cables) safe?						
3.3.1.13 Is the inhabitant able to use the washbasin safely (appropriate height)?						

**Bathtub**						
3.3.1.14 Is the inhabitant able to get in and out of the bathtub independently and safely?						
**Shower**						
3.3.1.15 Is the inhabitant able to enter and exit the shower independently and safely?						
**Toilets**						
3.3.1.16 Is the inhabitant able to sit down and get up independently from the toilets safely?						

Aids

Table - 3.3.2

For each item, please put a cross in the appropriate box. You also have the possibility to write a comment / clarification in the right column.

	Present and adequate	Present but requires adaptation	Not present and not needed	Not present, but necessary and to be installed	Not present but necessary and needs further assessment	Comment / clarification
**General information**						
3.3.2.1 Device for sitting in the bathroom						
**Bathtub**						
3.3.2.2 Bar / handle for the inhabitant to hold on to when entering / exiting the bathtub						
3.3.2.3 Device for sitting in the bathtub						
3.3.2.4 Anti-slip material on the bathtub floor						
**Shower**						
3.3.2.5 Bar / handle for the inhabitant to hold on to when entering / exiting the shower						
3.3.2.6 Device for sitting in the shower						
3.3.2.7 Anti-slip material on the shower floor						
**Toilets**						
3.3.2.8 Bar / handle for the inhabitant to hold on to when sitting / standing up from the toilets						
3.3.2.9 Booster seat for toilets						

3.3.3 Comment(s) / clarification(s) on the category “Bathroom” :

....

....

....

3.4 Bedroom

Environment

Table - 3.4.1

For each item, please put a cross in the appropriate box. You also have the possibility to write a comment / clarification in the right column.

	Yes	No. A solution must be put in place	No, but not a problem	Requires in-depth assessment	Not applicable	Comment / clarification
3.4.1.1 Is the bedroom door wide enough to allow the inhabitant to pass through with an aid (such as a rollator or wheelchair)?						
3.4.1.2 Is the inhabitant able to step over the threshold of the bedroom independently and safely?						
3.4.1.3 Is the bedroom sufficiently lit for the inhabitant to move around safely?						
3.4.1.4 Is the inhabitant able to manage the light sources in the bedroom in a safe way?						
3.4.1.5 Does the inhabitant have the possibility to manage at least one light source from the bed?						
3.4.1.6 Is the bedroom space large enough for the inhabitant to move around freely, potentially with an aid?						
3.4.1.7 Is the bedroom space free of clutter (allowing the inhabitant to move around safely)?						
3.4.1.8 Is the bedroom large enough for one person to help the inhabitant with his or her care in it?						
3.4.1.9 Is the bedroom large enough to accommodate the necessary care equipment (including a medical bed)?						
3.4.1.10 If there are mats in the bedroom, are they fixed to the floor to reduce the risk of falling?						
3.4.1.11 Is all bedroom furniture stable?						
3.4.1.12 Is the electrical equipment in the bedroom (sockets and cables) safe?						
3.4.1.13 Is the inhabitant's bed adapted to his/her needs and abilities (adequate height, adequate mattress)?						
3.4.1.14 Is the inhabitant's bed adapted to the needs of any carers (adequate location, adequate height)?						
3.4.1.15 Is it possible for the inhabitant to use a telephone from bed?						
3.4.1.16 Is the path between the bedroom and the bathroom sufficiently lit for the inhabitant to use it safely?						

3.4.1.17 Is the inhabitant able to manage the light sources on the path between the bedroom and the bathroom safely?						

Equipment present

Table 3.4.2

Please put a cross in the appropriate box. If necessary, you can also write a comment / clarification in the right column.

	Present and adequate	Present but requires adaptation	Not present and not needed	Not present, but necessary and to be installed	Not present but necessary and needs further assessment	Comment / clarification
3.4.2.1 Electric bed						
3.4.2.2 Side rail for bed						
3.4.2.3 Toilet chair						

3.4.3 Comment(s) / clarification(s) on the category “Bedroom” :

....

....

....

3.5 Interior staircase

Environment

3.5.1 Number of steps: ....

Table 3.5.2

For each item, please put a cross in the appropriate box. You also have the possibility to write a comment / clarification in the right column.

	Yes	No. A solution must be put in place	No, but not a problem	Requires in-depth assessment	Not applicable	Comment / clarification
3.5.2.1 Is the staircase free of clutter (allowing the inhabitant to use it safely)?						
3.5.2.2 Is the staircase sufficiently lit for the inhabitant to use it safely (sufficient light and the ability to switch on the staircase light source from the top and bottom)?						
3.5.2.3 Is the inhabitant able to manage the light sources in the staircase safely?						
3.5.2.4 Is the edge of the steps sufficiently visible for the inhabitant to use the stairs safely?						
3.5.2.5 Does the width of the steps allow the inhabitant to use the stairs safely?						

Aids

Table 3.5.3

For each item, please put a cross in the appropriate box. If necessary, you can also write a comment / clarification in the right column.

	Present and adequate	Present but requires adaptation	Not present and not needed	Not present, but necessary and to be installed	Not present but necessary and needs further assessment	Comment / clarification
3.5.3.1 Ramp on the left as you go up						
3.5.3.2 Ramp on the right as you go up						

3.5.4 Comment(s) / clarification(s) on the category “Interior staircase”:

....
....
....

3.6 Entrance hall

Environment

Table 3.6.1

For each item, please put a cross in the appropriate box. You also have the possibility to write a comment / clarification in the right column.

	Yes	No. A solution must be put in place	No, but not a problem	Requires in-depth assessment	Not applicable	Comment / clarification
**General information**						
3.6.1.1 Is the entrance hall sufficiently lit for the inhabitant to move around safely?						
3.6.1.2 Is the inhabitant able to manage the light sources in the entrance hall safely?						
3.6.1.3 Is the entrance hall large enough for the inhabitant to move around freely, potentially with an aid (such as a rollator or wheelchair)?						
3.6.1.4 Is the entrance hall large enough to accommodate a seating device if required?						
3.6.1.5 Is the entrance hall free of clutter (allowing the inhabitant to move around safely)?						
3.6.1.6 If there are mats in the entrance hall, are they fixed to the floor to reduce the risk of falling?						

3.6.2 Comment(s) / clarification(s) on the category “Entrance hall”:

....
....
....

3.7 Living room

Environment

Table 3.7.1

For each item, please put a cross in the appropriate box. You also have the possibility to write a comment / clarification in the right column.

	Yes	No. A solution must be put in place	No, but not a problem	Requires in-depth assessment	Not applicable	Comment / clarification
3.7.1.1 Is the door to the living room wide enough to allow the inhabitant to pass through with an aid (such as a rollator or wheelchair)?						
3.7.1.2 Is the inhabitant able to step over the threshold of the living room independently and safely?						
3.7.1.3 Is the living room sufficiently lit for the inhabitant to move around safely?						
3.7.1.4 Is the inhabitant able to manage the light sources in the living room safely?						
3.7.1.5 Is the living room space large enough for the inhabitant to move around freely, potentially with an aid?						
3.7.1.6 Is the living room space free of clutter (allowing the inhabitant to move around safely)?						
3.7.1.7 If there are mats in the living room, are they fixed to the floor to reduce the risk of falling?						
3.7.1.8 Is the inhabitant able to access the living room storage spaces independently and safely?						
3.7.1.9 Is all the furniture in the living room stable?						
3.7.1.10 Is the living room's electrical equipment (sockets and cables) secure?						
3.7.1.11 Does the inhabitant have a sitting device from which he/she can independently and safely get up in the living room?						

3.7.2 Comment(s) / clarification(s) on the category “Living room”:

………………………………………………...

....

....

3.8 Laundry

Environment

3.8.1 Does the inhabitant have to use a staircase to get to the laundry?

- o No
- o Yes

  If yes, please complete Annex II (found at the end of the tool)

Table - 3.8.2

For each item, please put a cross in the appropriate box. You also have the possibility to write a comment / clarification in the right column.

	Yes	No. A solution must be put in place	No, but not a problem	Requires in-depth assessment	Not applicable	Comment / clarification
3.8.2.1 Is the door to the laundry wide enough to allow the inhabitant to pass through with an aid (such as a rollator or wheelchair)?						
3.8.2.2 Is the inhabitant able to step over the threshold of the laundry independently and safely?						
3.8.2.3 Is the laundry sufficiently lit for the inhabitant to use it safely?						
3.8.2.4 Is the inhabitant able to manage the light sources in the laundry safely?						
3.8.2.5 Is the space in the laundry large enough for the inhabitant to move around freely, potentially with an aid?						
3.8.2.6 Is the space in the laundry large enough to accommodate a seating device if necessary?						
3.8.2.7 Is the laundry space free of clutter (allowing the inhabitant to move around safely)?						
3.8.2.8 Is the inhabitant able to access the laundry independently and safely?						
3.8.2.9 Is the inhabitant able to use the washing machine independently and safely?						
3.8.2.10 Is the inhabitant able to use the drying machine independently and safely?						

3.8.3 Comment(s) / clarification(s) on the category “Laundry”:

....

....

....

3.9 Cellar

Environment

3.9.1 Does the inhabitant have to use a staircase to get to the cellar?

- o No
- o Yes

  If yes, please complete Annex II (found at the end of the tool)

Table 3.9.2

For each item, please put a cross in the appropriate box. You can also leave a comment / clarification in the right column.

	Yes	No. A solution must be put in place	No, but not a problem	Requires in-depth assessment	Not applicable	Comment / clarification
3.9.2.1 Is the cellar door wide enough to allow the inhabitant to pass through with an aid (such as a rollator or wheelchair)?						
3.9.2.2 Is the inhabitant able to step over the cellar threshold independently and safely?						
3.9.2.3 Is the cellar sufficiently lit for the inhabitant to use it safely?						
3.9.2.4 Is the inhabitant able to manage the light sources in the cellar safely?						
3.9.2.5 Is the cellar space large enough for the inhabitant to move around freely, potentially with an aid?						
3.9.2.6 Is the cellar space free of clutter (allowing the inhabitant to move around safely)?						
3.9.2.7 Is the inhabitant able to access cellar storage independently and safely?						

3.9.3 Comment(s) / clarification(s) on the category “Cellar”:

....

....

....

3.10 Garage

Environment

3.10.1 Does the inhabitant have to use a staircase to get to the garage?

- No
- Yes

  If yes, please complete Annex II (found at the end of the tool)

Table 3.10.2

For each item, please put a cross in the appropriate box. You also have the possibility to write a comment / clarification in the right column.

	Yes	No. A solution must be put in place	No, but not a problem	Requires in-depth assessment	Not applicable	Comment / clarification
3.10.2.1 Is the garage door wide enough for the inhabitant to pass through it with an aid (such as a rollator or wheelchair)?						
3.10.2.2 Is the inhabitant able to step over the garage threshold independently and safely?						

3.10.2.3 Is the garage sufficiently lit for the inhabitant to move around safely?						
3.10.2.4 Is the inhabitant able to manage the light sources in the garage safely?						
3.10.2.5 Is the garage space free of clutter (allowing the inhabitant to move around safely)?						
3.10.2.6 Is the inhabitant able to open and close the door leading from the garage to the outside independently and safely?						

3.10.3 Comment(s) / clarification(s) on the category "Garage":

....

....

....

3.11 Balcony / Terrace

Environment

Table 3.11.1

For each item, please put a cross in the appropriate box. You also have the possibility to write a comment / clarification in the right column.

	Yes	No. A solution must be put in place	No, but not a problem	Requires in-depth assessment	Not applicable	Comment / clarification
3.11.1.1 Is the door from the home to the balcony/terrace wide enough to allow the inhabitant to pass through with an aid (such as a rollator or wheelchair)?						
3.11.1.2 Is the inhabitant able to step over the doorway from the home to the balcony/terrace independently and safely?						
3.11.1.3 Is the balcony/terrace space free of clutter (allowing the inhabitant to move around safely)?						

3.11.2 Comment(s) / clarification(s) on the category "Balcony / Terrace":

....

....

....

4. TOOLS AND MEANS AT THE INHABITANT'S DISPOSITION

4.1 - Table

For each tool/means mentioned, please put a cross in the appropriate box. If the tool is present and adequate but its use is problematic, please specify this in the right column.

**Possession of a / of:**	A. Yes and adequate	B. Yes, but to be adapted	C. No and not necessary	D. No but to be organised	Comment / clarification
Means / tools of communication					
4.1.1 Fixed telephone					
4.1.2 Mobile phone					
4.1.3 Internet connection					
4.1.4 Remote alarm					
Tools to help with activities of daily living					
4.1.5 Clamp for picking up objects from the ground					
4.1.6 Dressing aids (e.g., zip hook, button threader, sock threader)[4]					
4.1.7 Assistive feeding tools (e.g., adapted fork, adapted plate)[4]					
4.1.8 Transfer aids (e.g., transfer board, stork) [4]					

4.2 Other tool(s)/means available to the inhabitant:

....

4.3 Other tool(s)/means to be organised :

....

4.4 Comment(s) / clarification(s) on the category "Tools and means at the inhabitant's disposition" :

....
....
....

[4] If yes, please specify in the right column which tool(s) it is.

Annex I - Stairs to the home

Environment

Number of steps: ....

Presence of a turn in the staircase:

- o Yes
- o No

For each item, please put a cross in the appropriate box. You also have the possibility to write a comment / clarification in the right column.

	Yes	No. A solution must be put in place	No, but not a problem	Requires in-depth assessment	Not applicable	Comment / clarification
Is the staircase free of clutter (allowing the inhabitant to use it safely)?						
Is the staircase sufficiently lit for the inhabitant to use it safely (sufficient light and the ability to switch on the staircase light source from the top and bottom)?						
Is the inhabitant able to manage the light sources in the staircase safely?						
Is the edge of the steps visible enough for the inhabitant to use the stairs safely?						
Does the width of the steps allow the inhabitant to use the stairs safely?						

Aids

For each item, please put a cross in the appropriate box. If necessary, you can also write a comment / clarification in the right column.

	Present and adequate	Present but requires adaptation	Not present and not needed	Not present, but necessary and to be installed	Not present but necessary and needs further assessment	Comment / clarification
Ramp on the left as you go up						
Ramp on the right as you go up						

Comment(s) / clarification(s) on the category “Stair to the home”:

....

....

....

Annex II - Interior staircase to laundry / garage / cellar

Environment

Number of steps: ....

Presence of a turn in the staircase:

- Yes
- No

For each item, please put a cross in the appropriate box. You also have the possibility to write a comment / clarification in the right column.

	Yes	No. A solution must be put in place	No, but not a problem	Requires in-depth assessment	Not applicable	Comment / clarification
Is the staircase free of clutter (allowing the inhabitant to use it safely)?						
Is the staircase sufficiently lit for the inhabitant to use it safely (sufficient light and the ability to switch on the staircase light source from the top and bottom)?						
Is the inhabitant able to manage the light sources in the staircase safely?						
Is the edge of the steps visible enough for the inhabitant to use the stairs safely?						
Does the width of the steps allow the inhabitant to use the stairs safely?						

Aids

For each item, please put a cross in the appropriate box. If necessary, you can also write a comment / clarification in the right column.

	Present and adequate	Present but requires adaptation	Not present and not needed	Not present, but necessary and to be installed	Not present but necessary and needs further assessment	Comment / clarification
Ramp on the left as you go up						
Ramp on the right as you go up						

Comment(s) / clarification(s) on the category “Interior staircase to laundry / garage / cellar”:

....

....

....
